# The severity of ADHD and eating disorder symptoms: a correlational study

**DOI:** 10.1186/1471-244X-13-44

**Published:** 2013-02-01

**Authors:** Niklaus Stulz, Urs Hepp, Céline Gächter, Chantal Martin-Soelch, Anja Spindler, Gabriella Milos

**Affiliations:** 1 Psychiatric Services Aargau AG, Haselstrasse 1, CH-5401, Baden, Switzerland; 2 Cantonal Hospital Aarau, Tellstrasse 15, CH-5001, Aarau, Switzerland; 3 Department of Psychiatry, University Hospital Zurich, Culmannstrasse 8, CH-8091, Zurich, Switzerland

## Abstract

**Background:**

Attention deficit/hyperactivity disorders (ADHD) and eating disorders (ED) share several clinical features. Research on the association between ADHD and ED is still quite sparse and findings are ambiguous.

**Methods:**

Correlations between the severity of ADHD key features (Barratt Impulsiveness Scale, and Attention Deficit/Hyperactivity Disorder-Self-Rating questionnaire) and the severity of specific ED symptoms (Structured Interview for Anorexia and Bulimia Nervosa) were examined in 32 female patients diagnosed with ED.

**Results:**

Most correlations between the severity of ADHD features and the severity of ED symptoms were low (*r*<0.30) and did not reach statistical significance. The only exception was a statistically significant, but counterintuitive association between impulsivity and the avoidance of fattening food.

**Conclusions:**

The findings in this small sample suggest a weak link between the severity of ADHD key features and the severity of single ED symptoms in female patients with ED. The role of ADHD features for the development, maintenance, and treatment of EDs seems to be intricate and requires further study.

## Background

The three key features of attention deficit/hyperactivity disorders (ADHD)–inattention, hyperactivity, and impulsivity–are often present in individuals with eating disorders (ED) [1]. Core features of bulimia nervosa (BN) include binge eating and purging–behaviors that can be described as impulsive. In fact, impulsiveness has been shown to be increased in individuals with ED, particularly in individuals with binge–eating behavior, when compared to healthy controls [2,3]. Individuals with anorexia nervosa (AN) or BN also showed significant attention impairment in neuropsychological testing compared to healthy controls [4]. Last but not least, excessive exercise, which is typical in AN patients, may be considered a restless or hyperactive behavior.

In line with these findings, case-controlled studies found increased prevalence rates of ED in general, and of BN in particular, in ADHD samples compared with control groups (11–16% vs. 2–5%) [5-9]. Further studies without control groups reported very similar ED rates in individuals with ADHD [10-12]. Conversely, Wentz et al. [13] found an ADHD prevalence rate of 10–17% in ED patients, all of them with AN purging type. Although there was no control group, findings of this study suggest that the rate of ADHD may be increased in ED patients. The presence of comorbid ADHD in ED patients may affect the course of illness and thus may be clinically highly relevant for the treatment of ED.

However, whether ADHD is really increased in ED is still part of the debate. Study findings vary considerably. Contrasting the findings of Wentz et al. [13], some more recent studies reported ADHD rates between 3 and 9% in ED inpatients, suggesting that ADHD might be not or only slightly increased in these patients [14,15]. Limitations of these studies on the comorbidity of ED and ADHD relate to the populations used and to diagnostic parameters.

Concerning the subtypes of ED, recent longitudinal research has shown that, whereas the overarching category of ED is relatively stable over time, longitudinal crossover between the ED diagnoses (AN, BN, and ED not otherwise specified [EDNOS]) and between AN subtypes (binge eating/purging AN and restrictive AN) is common and recurrent [16-20]. Such diagnostic flux between specific ED diagnoses over the course of the illness suggests that there might be common biological and psychological causal and maintaining processes in different specific ED. This casts doubt on the existing scheme for classifying ED, which generates a disproportionate number of EDNOS [21]. Combined with shared clinical features among ED [22], the frequent diagnostic flux between ED diagnoses rather strengthens the view that ED might be best viewed as a single entity [23]. Such a “transdiagnostic” view may be clinically more useful and more appropriate for the examination and the understanding of ED [24].

Although ADHD has recently received more attention in adult psychiatry, research on the association between ADHD and ED is still quite sparse and ambiguous. Diagnostic criteria of ADHD varied widely in previous studies and few of them distinguished between symptoms of ADHD (such as inattention) and ADHD diagnosis [1]. Moreover, available research either focused on the prevalence of ADHD diagnoses in ED individuals and vice versa [5-7,13,14], or was concerned with the degree of impulsivity in patients with ED [2,25-27]. The only studies that looked at associations between ADHD and ED on the level of the severity of specific symptoms were restricted to either the ADHD feature of impulsivity or to BN patients [28-31].

The purpose of this study was to systematically examine the associations between the severity of the three ADHD key features (attention deficit, hyperactivity, and impulsivity) and the severity of ED symptoms and characteristics in individuals with any ED diagnosis in order to provide a more general view on the relationship between ADHD and ED symptoms by adopting a transdiagnostic view on ED.

## Methods

### Participants

This study included *N=*32 women diagnosed with a current DSM-IV [32] ED. Participants were consecutively recruited from the ED Unit of the University Hospital of Zurich. All participants gave informed consent. The study protocol was approved by the ethics committee of the Canton of Zurich, Switzerland, and carried out in compliance with the Helsinki Declaration.

Of the participants, 12 (37.5%) were diagnosed with AN, 6 (18.8%) with BN, and 14 (43.7%) with EDNOS. Seven (58.3%) of the AN cases were of the restrictive type, 5 (41.7%) of the binge/purging type. All BN cases were of the binge/purging type.

Eleven (34%) participants had at least one current comorbid axis I disorder, with anxiety (25%) and depressive (13%) disorders being most frequent. The number of ED patients diagnosed with comorbid ADHD varied between 0 and 28%, depending on the cut-off criteria used in the *Attention Deficit/Hyperactivity Disorder-Self-Rating* (ADHD-SR) screening questionnaire (see below) [33]. Eighteen participants (56%) were diagnosed with at least one axis II disorder, with depressive (31%), obsessive-compulsive (19%), and avoidant (16%) personality disorders being most frequent.

The age of the participants ranged between 17 and 46 years (*M=*23.1, *SD=*5.6). The mean age at ED onset was 17.1 (*SD=*4.0) years. At study entry, the participants had on average suffered from ED for 6.0 (*SD=*4.6) years. The majority of the participants were not married (91%), had no partner (59%), and were in part-time or full-time paid employment (53%). Almost half of the participants (44%) were still in education.

### Measures

All participants were examined using standardized clinical interviews and valid self-report questionnaires.

The *Structured Interview for Anorexia and Bulimia Nervosa-Expert Rating* (SIAB-EX) was developed for reliable and valid assessment of the specific and the general psychopathology of ED and provides a measure of the severity of ED symptoms [34]. The SIAB-EX covers a wide spectrum of symptoms frequently seen in different types of ED, ranging from body image disturbances, bulimic symptoms, social integration, and sexuality to depression, compulsion, and anxiety. Each of these symptoms is rated on a rank-ordered scale ranging from 0=no to 4=very severe/strong/frequent.

The *Attention Deficit/Hyperactivity Disorder-Self-Rating* (ADHD-SR) questionnaire is a German instrument for the diagnosis of ADHD in adults according to DSM-IV or ICD-10 [35]. Eighteen of the 22 items ask for symptoms of ADHD (e.g. I am fidgety). The items are rated on a 4-point scale (0=does not apply, 1=mild (occasionally present), 2=moderate (often present), 3=severe (almost always present)) and are grouped into three dimensions (psychometric scales) representing attention deficit, hyperactivity, and impulsivity. Four additional items deal with further criteria for the diagnosis of ADHD (e.g. suffering due to these symptoms). An ADHD symptom was considered to be present if its intensity was rated with at least 2 (“moderate”). Such strict cutoff is recommended for research settings. Less strict clinical criteria, however, already assume a symptom to be present if it is rated at least “mild (occasionally present)” [33]. Evaluation of the psychometric properties of the ADHD-SR revealed good internal consistencies of the sum scale and the subscales (.72-.90), high retest reliabilities (.78-.89), and good convergent validity [35].

The *Barratt Impulsiveness Scale, Version 11* (BIS-11) is a 30-item self-report measure to assess (1) motor impulsivity, (2) cognitive/attention impulsivity, and (3) non-planning impulsivity [36]. The German version of the BIS-11 has adequate internal consistency (Cronbach’s *alpha=*0.69) and differentiated well between psychiatric patients and control persons. However, the original factor structure was not unambiguously replicated in the German translation [37]. So we only considered the sum score of the BIS-11 in this study. The BIS has been used to assess impulsivity in ED patients in several previous studies [2,38,39].

### Statistical analyses

The associations between the degree of severity of ADHD core features and the severity of specific ED symptoms were examined using Pearson’s product-moment correlations (for continuous variables) and Spearman’s rank correlations (for rank-ordered variables). If an ED symptom was present in none or only one participant (e.g. enema use), no correlations were calculated. To control for type I error inflation when performing multiple tests in the same sample, we used Bonferroni corrected alpha levels [40].

## Results

Many of the ED patients in our sample had at least subsyndromal ADHD. Although none of them was diagnosed with an ADHD when using the strict ADHD-SR research criteria [33], the proportion of participants with comorbid ADHD increased to 29% when using less strict clinical criteria [33]. Furthermore, the BIS-11 sum score suggested significantly higher impulsivity in our ED sample (*M = *62.7, *SD = *10.3) than among healthy German controls (*M = *51.2, *SD = *7.3; *t*(840)* = *8.585, *P <*0.001) (Table 1). Comparison of our ED participants with healthy controls in the three subscales of the ADHD-SR was not possible; the assessment of ADHD-SR norm data in a community sample is still in progress [41].

**Table 1:** Correlations between the severity of the three core features of the attention deficit/hyperactivity disorder and the severity of symptoms or characteristics of eating disorders (N=32)

				***r*(*p*)**			
**Eating disorder symptom/characteristic**	***n***^**a**^	***M*(*SD*)**	***Md***	**ADHD-SR: attention deficit scale**	**ADHD-SR: hyperactivity scale**	**ADHD-SR: impulsivity scale**	**BIS-11: sum score**
Age (years)^b^	32	23.1 (5.6)		−0.152 (0.406)	−0.230 (0.205)	−0.035 (0.847)	−0.118 (0.519)
Age at onset (years)^b^	32	17.1 (4.0)		−0.011 (0.952)	−0.004 (0.984)	0.133 (0.467)	0.312 (0.082)
Current BMI (kg/m^2^)^b^	32	17.1 (3.8)		0.119 (0.515)	−0.185 (0.311)	0.107 (0.561)	0.395 (0.025)
Lowest BMI (kg/m^2^)^b^	32	14.8 (3.0)		0.109 (0.551)	−0.170 (0.353)	0.079 (0.667)	0.531 (0.002)
Highest BMI (kg/m^2^)^b^	32	22.2 (3.7)		0.248 (0.171)	−0.075 (0.684)	0.265 (0.143)	0.352 (0.048)
Weight phobia^c^	32		2.0	0.202 (0.267)	0.168 (0.357)	0.323 (0.071)	0.302 (0.093)
Dependence of self-esteem on body shape^c^	32		2.0	0.367 (0.039)	0.070 (0.705)	0.279 (0.122)	0.062 (0.738)
Body-image disturbances^c^	32		3.0	0.344 (0.054)	0.332 (0.063)	0.064 (0.728)	0.370 (0.037)
Denial of seriousness of low body weight^c^	23		2.0	0.393 (0.063)	0.209 (0.339)	0.052 (0.813)	0.081 (0.712)
Periods (last 3 months)^c^	29		2.0	−0.028 (0.885)	−0.065 (0.739)	−0.160 (0.406)	−0.435 (0.018)
Severity of binge eating episodes^c^	32		2.0	−0.050 (0.787)	−0.249 (0.169)	0.171 (0.350)	0.218 (0.231)
Sense of lack of control^c^	18		2.0	0.137 (0.588)	0.300 (0.227)	0.512 (0.030)	0.430 (0.075)
Self-induced vomiting^c^	32		1.0	−0.159 (0.385)	−0.203 (0.265)	0.232 (0.202)	0.252 (0.163)
Laxative abuse^c^	32		0.0	0.269 (0.136)	0.107 (0.558)	0.143 (0.434)	0.504 (0.003)
Diuretics^c^	32		0.0	--- ^d^	--- ^d^	--- ^d^	--- ^d^
Appetite suppressants^c^	32		0.0	--- ^d^	--- ^d^	--- ^d^	--- ^d^
Medication to increase thyroid metabolism^c^	32		0.0	--- ^d^	--- ^d^	--- ^d^	--- ^d^
Neglect of insulin treatment^c^	32		0.0	--- ^d^	--- ^d^	--- ^d^	--- ^d^
Excessive fasting^c^	32		0.0	0.177 (0.334)	0.176 (0.335)	−0.093 (0.614)	0.415 (0.018)
Excessive exercise^c^	32		1.0	0.119 (0.518)	0.137 (0.455)	0.003 (0.988)	0.220 (0.225)
Enemas^c^	32		0.0	--- ^d^	--- ^d^	--- ^d^	--- ^d^
Use of vomitives^c^	32		0.0	--- ^d^	--- ^d^	--- ^d^	--- ^d^
Frequency of binge eating episodes^c^	32		1.0	−0.037 (0.839)	−0.184 (0.314)	0.220 (0.225)	0.213 (0.243)
Frequency of inappropriate compensatory behaviors^c^	32		1.0	−0.020 (0.914)	−0.168 (0.357)	0.108 (0.556)	0.214 (0.240)
Dieting or fasting^c^	32		2.0	0.394 (0.026)	0.329 (0.066)	0.312 (0.082)	0.226 (0.214)
Avoidance of fattening food^c^	32		2.0	0.244 (0.177)	0.301 (0.094)	**0.583 (0.000)**	0.390 (0.028)
Limits for caloric intake^c^	32		0.5	0.273 (0.131)	0.377 (0.034)	0.439 (0.012)	0.259 (0.152)
Preoccupation with body slimness^c^	32		2.5	0.447 (0.010)	0.271 (0.134)	0.311 (0.083)	0.411 (0.020)
*M* (*SD*)				7.3 (4.3)	6.3 (3.2)	3.0 (3.1)	62.7 (10.3)

BMI=Body Mass Index, ADHD-SR=Attention Deficit Hyperactivity Disorder–Self-Report, BIS-11=Barratt Impulsiveness Scale, Version 11.

Bonferroni corrected alpha=0.05/88=0.00057. Values in boldface are significant correlations when applying Bonferroni correction.

^a^Some items were not applicable for all patients in the sample. For example, the item “Denial of seriousness of low body weight” was not applicable for 9 patients who were not underweight.

^b^Pearson’s product–moment correlation.

^c^Spearman’s rank correlation. The items of the Structured Interview for Anorexia and Bulimia Nervosa–Expert Rating (SIAB–EX) were rated on a rank-ordered scale ranging from 0=no to 4=very severe/strong/frequent.

^d^Symptom or characteristic was reported by none or only one patient. No correlations were calculated.

As is shown in Table 1, the majority of the correlations between the severity of ADHD core features and the severity of specific ED symptoms or characteristics were low (*r <*0.30) [42], indicating only weak associations between ADHD and ED symptoms, if any. Even if one is using a conventional alpha level of .05, only relatively few correlations reached statistical significance (Table 1). When adopting a Bonferroni corrected critical alpha level of 0.05/88=0.00057 to control for the inflated type I error due to the testing of 88 correlations in the sample, the only correlation reaching statistical significance was between the degree of impulsivity as measured by the ADHD-SR and more severe avoidance of fattening food. Impulsivity as measured by the BIS-11, however, turned out to be not significantly associated with the severity of such anorectic behavior. The correlation of impulsivity as measured by the BIS-11 and of impulsivity as measured by the ADHD-SR was low (*r = *0.28, *P = *0.12), suggesting that the two scales measure different aspects of impulsivity.

Finally, additional analyses within the specific ED subtypes in our sample (AN-restrictive type, AN-binge/purging type, BN-binge/purging type, and EDNOS) also revealed no significant correlations between ADHD and ED symptoms when controlling for inflated type I error due to multiple testing. (The corresponding results are not shown here due to the small sizes of the subsamples and due to space limitations). Furthermore, the ED subtypes did not differ significantly on the ADHD-SR scales either (attention deficit: *F *(3,28) = 2.087; *P = *0.125; hyperactivity: *F *(3,28) = 0.818; *P = *0.495; impulsivity: *F *(3,28) = 2.246; *P = *0.105) and in the BIS sum score (*F *(3,28) = 1.778; *P = *0.174).

## Discussion

This study did not find more severe ADHD characteristics to be associated with more severe ED symptoms in women diagnosed with ED. Although about one fourth of the sample presented at least subsyndromal ADHD, neither the severity of inattention nor the severity of hyperactivity was associated with any of the ED symptoms’ severity. The only statistically significant correlation (when controlling for inflated type I error due to multiple testing in the sample) was found between the degree of impulsivity and avoidance of fattening food. In addition to the preponderance of these small and non-significant correlations between the severity of ADHD and ED features, the observed associations were often counterintuitive; positive correlations were found where negative correlations were expected and vice versa. For instance, higher impulsivity was not only statistically significant related to the avoidance of fattening food, but it also tended to be positively correlated with limiting caloric intake, dieting, and excessive fasting. These behaviors all require strict discipline and thus seem not to be very compatible with impulsivity. These preliminary findings, though unexpected, could elucidate an important peculiarity of ED: the extreme control, which is a central symptom of AN patients (e.g., when encountering fattening food) could be interpreted as an answer to a primary impulsivity, and explain the frequent diagnostic cross over from AN to BN [16-20]. Although we usually associate impulsivity with BN, a high prevalence of ADHD was also found in a sample with mainly AN subjects [13].

Whereas our findings suggest that impulsivity might be increased in ED patients compared to healthy controls [2,3], it does not appear to be a predictor of the severity of eating-related impulsive behavior such as binge eating or purging, though one would expect impulsivity to abet sudden decisions to binge and purge. Interestingly, such lack of an association between impulsivity and ED symptom severity has already been reported in previous studies among BN patients [27,28].

Given the examination of few (*N = *32) patients in our study, one could suppose that the lack of significant correlations is related to the low statistical power in such a small sample. However, the correlation coefficients, which (unlike significance tests) constitute a measure that is independent of the sample size, in general indicated only small associations between the severity of ADHD and ED characteristics (most *r*’s < 0.30). For instance, the highest correlation between an indicator of impulsivity (the BIS-11 sum score) and a measure of binge eating (the severity of binge eating episodes) equaled *r = *0.218. That is, knowing BIS-11 impulsivity scores enables explanation of less than 5% (*r*^*2*^* = *0.218^2^ = 0.048) of the variance of the severity of binge eating episodes. It is therefore unlikely that impulsivity scores simply reflect the presence or absence of impulsive eating patterns. In line with this, Davies et al. [38] did not find higher BIS total scores in BN patients when compared to AN patients. To provide yet another example, there was also no substantial association between hyperactivity and excessive exercise (*r = *0.137), which was also contrary to our expectations.

Considering these unexpected findings, one could argue that adopting a “transdiagnostic” view on ED–and hence aggregating results across different specific ED diagnoses–may have masked associations between ADHD features and ED symptoms in specific ED types. Correlational analyses within the specific ED subtypes in our sample (AN-restrictive type, AN-binge/purging type, BN-binge/purging type, and EDNOS), however, did not provide any indication of ADHD-ED symptom severity relations in specific ED subtypes. Furthermore, though longitudinal diagnostic flux between specific ED diagnoses is frequent and recurrent, the overreaching diagnosis of ED and the characteristics of ADHD are both relatively stable over time [17-19]. If there were striking associations between ADHD features and the severity of ED symptoms, one might expect them to be revealed even in a diagnostically mixed sample.

In summary, the findings of this study indicate that the associations between the severity of ADHD features and the severity of specific ED symptoms are low in women diagnosed with ED. This is in line with some previous studies that failed to find increased rates of ADHD in ED [14,15]. And even if there should be an increased prevalence of ADHD in ED individuals [5-7,13], the relation between these two disorders might be less straightforward and more complex than expected based on clinical features shared by both disorders. Therefore, regarding clinical implications, if ADHD key features are salient in ED, both symptomatologies seem to require equal clinical attention. Focusing primarily on ADHD symptoms and expecting that ED symptoms will be automatically alleviated by such intervention might result in disappointing outcomes. Several case reports suggested that methylphenidate and other psychostimulants may be effective in reducing BN symptoms in patients with comorbid ADHD and BN [43-46]. These case reports should be interpreted with caution since adequate rating scales to evaluate improvement in bulimic symptoms were not used in the majority of them. Some of the reports included patients who were diagnosed not only with BN and ADHD, but with cluster B personality disorders as well. In our sample only two participants had such personality disorder. Therefore, the lack of an association between ADHD features and the severity of ED symptoms observed in our study calls into question the promise of interventions targeting on ADHD features as a treatment for ED symptomatology.

Among the limitations of this study are the small sample size and the aggregation across specific ED, both of which were discussed earlier. The problem of the relatively small number of patients in this study is accentuated when performing analyses within specific ED subtypes. Thus, our null-findings resulting from subgroup analyses should be treated with caution. They maybe just reflect the need for larger samples. Further research is needed to examine associations between the severity of specific ADHD and ED symptoms in ED subtypes.

As a further potential limitation of this study, we used self-report questionnaires (ADHD-SR and BIS-11) to assess ADHD characteristics. The *Structured Clinical Interview for DSM-IV Disorders*[47], which is the most common instrument to asses mental disorders, does not encompass ADHD. To date there is no standardized method for identifying subjects with ADHD [1], and existing interviews to assess neuropsychiatric disorders with onset during childhood are extremely time-consuming [13]. Nevertheless, clinicians’ ratings of the severity of ADHD core features might have resulted in different findings than the self-report questionnaires used in this study. Based on information from the ADHD-SD, none of our participants was diagnoses with an ADHD when applying the strict research criteria (an ADHD symptom was considered to be present if its intensity was rated at least “moderate” by the participant). However, when using the less strict clinical criteria (a symptom is already assumed to be present if it is rated at least “mild (occasionally present)” ), the proportion of ADHD diagnoses in our ED sample increased to 29%. Thus, at least subsyndromal ADHD was present in many patients of our sample [33]. Finally, normative data of the ADHD-SR questionnaire is not yet available for healthy controls. So it remains unclear whether our ED patients reported increased scores on the ADHD-SR scales compared to healthy controls. Future research on associations between ADHD and ED would benefit from efficient and thoroughly validated instruments to assess ADHD.

## Conclusions

Despite the small sample, our study suggests that ADHD key features such as impulsivity may be increased in individuals with ED, but that their severity does not play a crucial role regarding the severity of specific ED symptoms. Although ADHD is frequently observed in ED individuals, the relation between these two disorders seems to be complex at the level of specific symptoms and features. Further research is needed to elucidate the association between the severity of ADHD features and the severity of specific ED symptoms.

## Competing interests

The authors declare that they have no competing interests.

## Authors’ contributions

CG, CM, AS, and GM designed the study. CG was responsible for data collection. NS performed the statistical analyses. NS, UH, and GM were involved in the interpretation of the data. NS, UH, and GM drafted the manuscript. All authors reviewed the manuscript several times and have read and approved the final version of the manuscript.

## Pre-publication history

The pre-publication history for this paper can be accessed here:

http://www.biomedcentral.com/1471-244X/13/44/prepub
